# Structural basis for the toxic activity of MafB2 from maf genomic island 2 (MGI-2) in *N. meningitidis* B16B6

**DOI:** 10.1038/s41598-023-30528-9

**Published:** 2023-02-27

**Authors:** So Hyeon Park, Sun Ju Jeong, Sung Chul Ha

**Affiliations:** Beamline Department, Pohang Accelerator Laboratory, Pohang University of Science and Technology, Pohang, Gyeongbuk 37673 Republic of Korea

**Keywords:** Biochemistry, Structural biology

## Abstract

The Maf polymorphic toxin system is involved in conflict between strains found in pathogenic *Neisseria* species such as *Neisseria meningitidis* and *Neisseria gonorrhoeae*. The genes encoding the Maf polymorphic toxin system are found in specific genomic islands called maf genomic islands (MGIs). In the MGIs, the MafB and MafI encode toxin and immunity proteins, respectively. Although the C-terminal region of MafB (MafB-CT) is specific for toxic activity, the underlying enzymatic activity that renders MafB-CT toxic is unknown in many MafB proteins due to lack of homology with domain of known function. Here we present the crystal structure of the MafB2-CT_MGI-2B16B6_/MafI2_MGI-2B16B6_ complex from *N. meningitidis* B16B6. MafB2-CT_MGI-2B16B6_ displays an RNase A fold similar to mouse RNase 1, although the sequence identity is only ~ 14.0%. MafB2-CT_MGI-2B16B6_ forms a 1:1 complex with MafI2_MGI-2B16B6_ with a Kd value of ~ 40 nM. The complementary charge interaction of MafI2_MGI-2B16B6_ with the substrate binding surface of MafB2-CT_MGI-2B16B6_ suggests that MafI2_MGI-2B16B6_ inhibits MafB2-CT_MGI-2B16B6_ by blocking access of RNA to the catalytic site. An in vitro enzymatic assay showed that MafB2-CT_MGI-2B16B6_ has ribonuclease activity. Mutagenesis and cell toxicity assays demonstrated that His335, His402 and His409 are important for the toxic activity of MafB2-CT_MGI-2B16B6_, suggesting that these residues are critical for its ribonuclease activity. These data provide structural and biochemical evidence that the origin of the toxic activity of MafB2_MGI-2B16B6_ is the enzymatic activity degrading ribonucleotides.

## Introduction

Microorganisms have evolved strategies to obtain resources such as space and nutrients for their own survival. Simple strategies include killing other organisms or inhibiting their growth in the environment. The use of proteinaceous toxins is a typical example of restricting the growth of other organisms^1,2^. For example, lantibiotics, polypeptides containing lanthionine, are used by Gram-positive bacteria such as *Lactococcus lactis* to kill or inhibit other bacterial cells^3^. Recent reports have shown that a family of multidomain toxins called polymorphic toxins (PT) are involved in interstrain competition in the same species^4–6^. Polymorphic toxins are characterized by modular domain arrangement with an N-terminal module for transportation and a C-terminal module for toxicity. This family of PTs includes contact-dependent growth inhibition (CDI) systems, colicins and S-type pyocins, rearrangement hot spot (RHS) repeat-containing proteins and effectors of type VI secretion system such as VgrG and Hcp^7–13^. The MafB toxin in *Neisseria* species also belongs to PTs family.

The genes encoding MafB proteins are found in the specific loci called maf genomic islands (MGIs) on the chromosomes of pathogenic *Neisseria* spp. such as *Neisseria gonorrhoeae* (the gonoccocus) and *Neisseria meningitidis* (the meningococcus)^14^,. There are multiple types of MGIs, which are classified as MGI-1, MGI-2, MGI-3, MGI-4 and MGI-5, depending on the genes flanking the MGIs^15^. For example, MGI-1 is flanked by *anmK* (the gene for anhydro-N-acetylmuramic acid kinase) and an ORF that encodes a hypothetical periplasmic protein similar to the N-terminus of FfpC^14,15^. *N. gonorrhoeae* harbors the 5 types of MGIs, but *N. meningitidis* has only MGI-1, -2, and -3^15^.

MafA, which is known to function as an adhesion by binding to a specific glycolipid (gangliotetraosylceramide, GgO4), is found upstream of MafB^14–16^. A recent report showed that MafA located in the outer membrane (OM) is the transporter of MafB for the exposure of MafB on the extracellular surface of the OM^17^. It is assumed that the C-terminal domain (MafB-CT) of MafB on the OM is cleaved and transported to the target cells by an unknown mechanism^17^. The *mafB* gene is always immediately followed by a *mafI* gene that encodes a small polypeptide of approximately 100 amino acids^15,18^. Since MafB contains a specific domain (MafB-CT) in the C-terminal region that has toxic activity, a gene encoding an immunity protein, MafI, is necessary to protect MafB-producing cells from the toxic activity. Therefore MafB and MafI exist as MafBI modules in MGIs. As a feature of the polymorphic toxin system, several alternative MafBI modules encoding solely the C-terminal part of the toxin (MafB-CT) and its cognate MafI are found in MGIs downstream of the *mafBI* module encoding the full length toxin^15^. It is thought that the downstream alternative MafBI modules could serve used as alternative MafB toxins after genetic recombination. Alternative toxin production by recombination events has been reported^19^.

MafB toxin consists of a signal peptide, an N-terminal DUF1020 domain with unknown function and a C-terminal toxin domain^14,15^. A sequence motif between the N-terminal DUF1020 domain and the C-terminal domain is conserved in different MafB toxins, and is used to divide MafB toxins into 3 classes^15^. Class 1, 2, and 3 MafB contain a VSFDF motif with a bacterial intein-like (BIL) domain, a VKYDT motif and a DWVKN motif, respectively. The sequence of the N-terminal DUF1020 domain in the three MafB toxin classes is conserved well with more than ~ 85% sequence identity, but the C-terminal toxin domains displays sequence variation with less than 35% identity, suggesting that the same class of MafB toxins can have different toxic activities^14,15^. The C-terminal region has conserved domains such as bacterial toxin 50 (pfam15542, a predicted RNase), novel toxin 17 (pfam15524, a predicted RNase), MafB19-like deaminase (pfam14437) and bacterial EndoU nuclease (pfam14436, a demonstrated RNase)^15^; thus, this domain causes cell toxicity through enzyme activity. Specifically, when RNase activity is used in the toxic enzyme activity of PTs, various RNase enzymatic activities such as EndoU nuclease activity cleaving after uridylates of RNA^15^, RNase activity cleaving RNA non-specifically^20^, RNase activity specific to ribosomal RNA^21^ and tRNase activity specific to tRNA^22^ have been shown to be the basis of the toxic activity of the toxin domain.

An in silico analysis and subsequent experimental analysis using *Neisseria* spp. revealed the role of a novel polymorphic toxin system, MafB, in intrastrain competition and identified a number of MafB prototype proteins exhibiting a conserved N-terminal region but a highly variable C-terminal region^14,15^. However, it is known that the MafB-CT is the toxic domain that should have cell damaging activity. Although the toxic activity of the MafB-CT can be inferred in some cases by querying domain databases, there are still many MafB toxins for which the in silico analysis is not able to reveal the cause of toxicity. When the information obtained through sequence similarity investigation is limited, structural studies can provide additional insights into the enzymatic basis for toxicity. This is accomplished by searching for structural homologs with known enzymatic activity, because the overall protein structure rather than the sequences is conserved for similar biochemical functions^23^. For example, structural studies of the C-terminal domain (CdiA-CT^Ykris^) of CdiA from *Yersinia kristensenii*, which is involved in contact-dependent growth inhibition and is also a polymorphic toxin, showed that CdiA has a domain similar to RNase A and uses ribonuclease for toxic activity even though the sequence similarity is very low^20^. MafB2_MGI-2B16B6_ is a polymorphic toxin present in MGI-2 in *N. meningitidis* strain B16B6 and it has been shown that MafB2-CT_MGI-2B16B6_ has toxic activity^14^. Although the amino acid sequences suggest the presence of a conserved toxin domain predicted to adopt the BECR (Barnase-EndoU-ColicinE5/D-RelE) fold, further supporting experimental data are limited. Accordingly, in order to gain insight into the enzymatic activity governing the toxicity of the C-terminal domain of MafB2_MGI-2B16B6_, we have determined the X-ray crystal structure of a MafB2-CT_MGI-2B16B6_/MafI2_MGI-2B16B6_ complex at 1.5 Å resolution and delineated the residues important for the toxic activity of MafB2-CT_MGI-2B16B6_ by using mutagenesis and cell toxicity assays. The structural and biochemical studies indicate that MafB2-CT_MGI-2B16B6_ is similar to the RNase A superfamily.

## Results and discussion

### Structure determination

**Figure 1:**
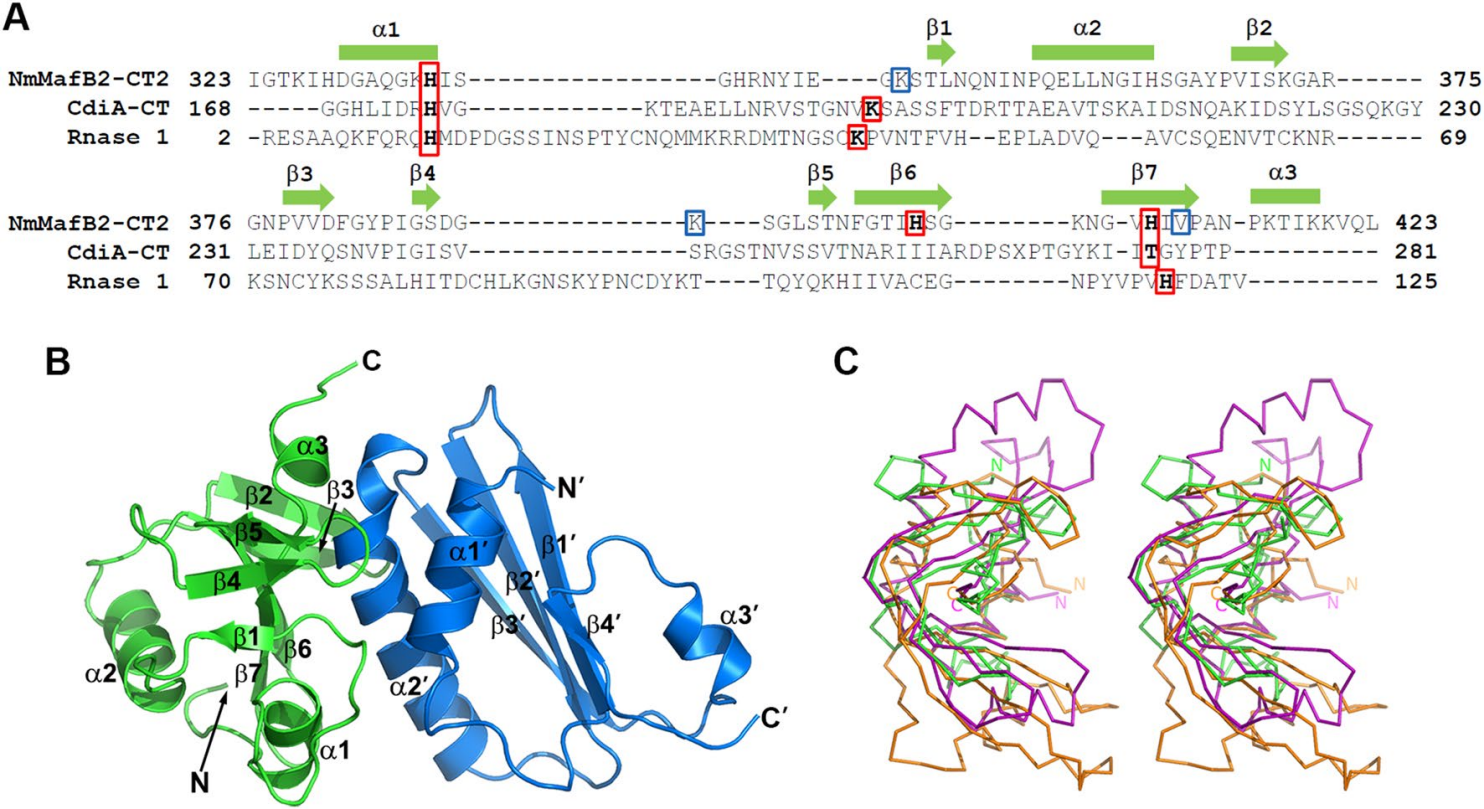
Overall structure of the NmMafB2-CT2/NmMafI2 complex. (**A**) Structure-guided sequence alignment of NmMafB2-CT2 with CdiA-CT^Ykris^ and RNase 1. The rectangles (α-helixes) and arrows (β-strands) in the above alignment indicate the secondary structures of NmMafB2-CT2. Putative catalytic residues are shown in red boxes, and the residues (K346, K391 and V411) near the catalytic residues of CdiA-CT^Ykris^ or RNase 1 but not critical for toxicity activity are in blue boxes. (**B**) Ribbon diagram of the NmMafB2-CT2/NmMafI2 complex. NmMafB2-CT2 and NmMafI2 are shown in green and sky blue, respectively. N- and C-termini, and the secondary structure elements are labeled. A prime symbol is added for NmMafI2. (**C**) Stereoview of the structural overlay of NmMafB2-CT2, CdiA-CT^Ykris^ (PDB code: 5E3E) and RNase 1 (PDB code: 3TSR). The ribbon diagrams of NmMafB2-CT2, CdiA-CT^Ykris^ and RNase 1 are displayed in green, magenta and orange, respectively. The view was rotated by ~ 90° horizontally from that of (**B**) with the upper part toward the viewer.

In order to infer the enzymatic properties contributing the toxic activity of MafB2_MGI-2B16B6_, which is the second MafB protein encoded in maf genomic island 2 (MGI-2) from *N. meningitidis* B16B6, the X-ray crystal structure of the complex between the toxin domain of MafB2_MGI-2B16B6_, hereafter referred to as NmMafB2-CT, and the corresponding immunity protein, MafI2_MGI-2B16B6_, hereafter referred to as NmMafI2, was determined at 1.5 Å resolution with 18.0% R_work_ and 20.8% R_free_ using a single anomalous dispersion method (Table 1). The toxin domain (Ala270-the C-terminus) of NmMafB2-CT and full-length NmMafI2 were used for the structural studies. There were no breaks in the polypeptide chains of NmMafB2-CT and NmMafI2 by poor electron density map. However, the N-terminal 53 residues from Ala270 to Ser322 of NmMafB2-CT were invisible probably due to the degradation of NmMafB2-CT during crystallization (Supplementary Fig. 1). The model of NmMafB2-CT contains residues from Ile323 to the C-terminus and that of NmMafI2 contains residues from Leu5 to Ile103. Hereafter, the model of NmMafB2-CT containing residues from Ile323 to the C-terminus is referred to as NmMafB2-CT2. Only one NmMafB2-CT2/NmMafI2 complex is found in the crystallographic asymmetry unit (Fig. 1B), suggesting that one NmMafI2 is required for counter-interaction with one NmMafB2-CT2, which was also consistent with the size exclusion chromatography results (data not shown). Notably, the sequence of the toxin domain of MafB2_MGI-2B16B6_ is identical to that of MafB2 encoded in maf genomic island 2 (MGI-2) from *N. meningitidis* NEM8013 and the corresponding immunity proteins are identical except two amino acids, suggesting that strains B16B6 and NEM8013 are not conflicted by the toxin domain of MafB2 (Supplementary Fig. 2)^14,15^.

**Table 1:** Data collection and refinement statistics of NmMafB2-CT2/NmMafI2 complex.

Statistics	Native	Se-Met
Data collection		
Space group	C2	C2
Cell dimensions (Å)		
a, b, c (Å)	78.69, 39.79, 65.55	79.28, 39.96, 67.22
α, β, γ (°)	90.0, 109.2, 90.0	90.0, 109.9, 90.0
Resolution (Å)	30–1.5 (1.53–1.5)	30–1.9 (1.93–1.9)
R_merge_^a^ (%)	6.5 (32.4)	14.8 (83.0)
CC_1/2_^b^	0.999 (0.970)	0.99 (0.882)
I/σ (I)	48.3 (9.0)	32.6 (6.6)
Completeness (%)	97.9 (97.3)	99..8 (100.0)
Redundancy	7.3 (7.4)	7.0 (7.0)
Wilson B factor (Å^2^)	13.26	15.0
Structure refinement		
Resolution (Å)	30–1.5	
Reflections, total/test set	30,286/2000	
R_work_^c^ / R_free_	18.0/20.8	
No. atoms, protein/water	1586/179	
R.m.s.deviation		
Bond lengths (Å)	0.006	
Angles (°)	0.808	
Average B-factor (Å^2^)		
Protein	15.2	
Ramachandran plot (%)		
Favored region	100.0	
Allowed region	0.0	

The numbers in parentheses are statistics from the highest resolution shell.

^a^*R*_merge_ = Σ |*I*_obs_—*I*_avg_| / *I*_obs_, where *I*_obs_ is the observed intensity of individual reflection and *I*_avg_ is average over symmetry equivalents. ^b^ CC_1/2_^24^.

^c^*R*_work_ = Σ ||*F*_o_| −|*F*_c_|| / Σ |*F*_o_|, where |*F*_o_| and |*F*_c_| are the observed and calculated structure factor amplitudes, respectively. *R*_free_ was calculated with 6.6% of the data.

### Overall fold

**Figure 2:**
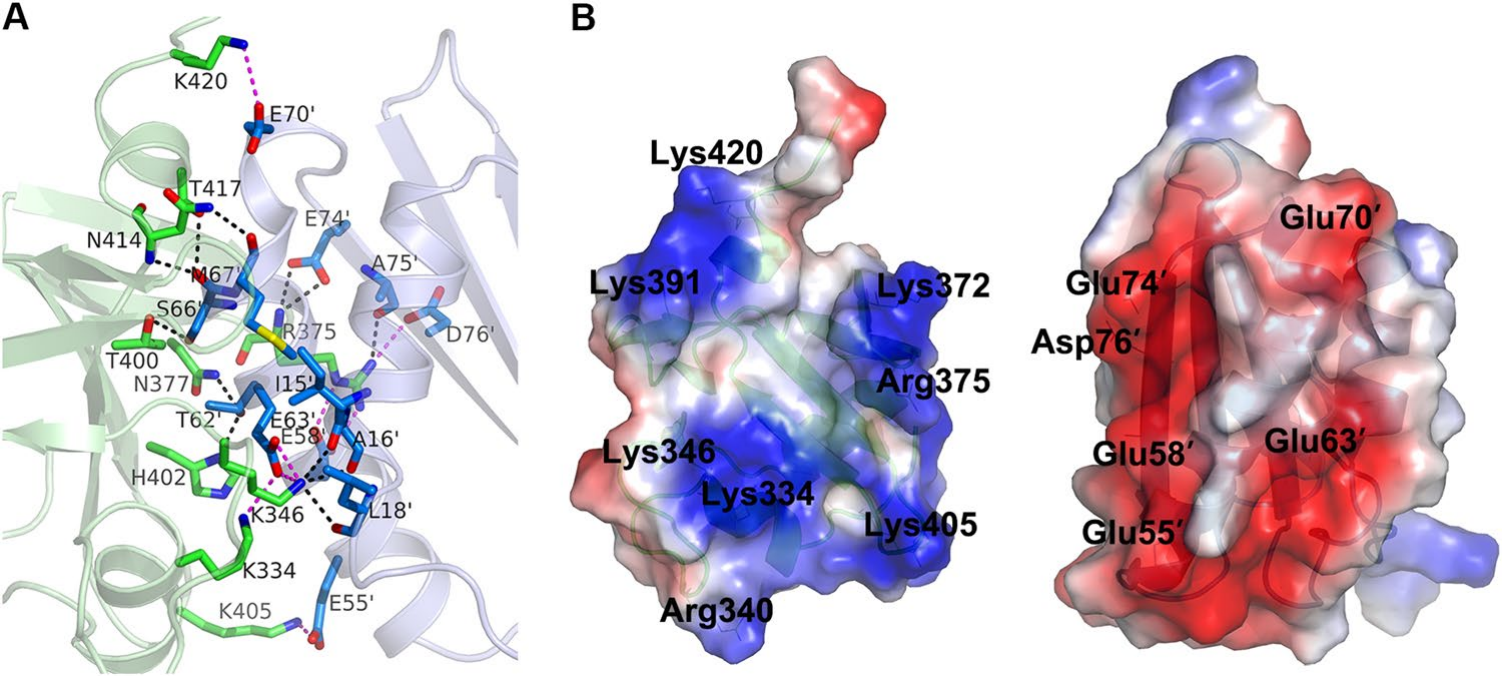
Interface between NmMafB2-CT2 and NmMafI2. (**A**) Detailed view of the interface between NmMafB2-CT2 and NmMafI2. The residues involved in hydrogen bond and salt bridge interactions are shown in the stick model in green (NmMafB2-CT2) and sky blue (NmMafI2). The hydrogen bonds and salt bridges are shown as dash lines in black and red, respectively. The residues are labeled, and those of NmMafI2 are indicated with a prime symbol (′). (**B**) Open book view of the NmMafB2-CT2/NmMafI2 complex in a surface model with the electrostatic potentials. The potentials are shown in the range of − 75 kT/e (red, negative potential) to + 75 kT/e (blue, positive potential). The positively charged surface of NmMafI2 counteracts with the negatively charged surface of NmMafB2-CT2 in the interface of the complex. The positions of positively charged residues on the surface of NmMafB2-CT2 and negatively charged residues on the surface of NmMafI2 are indicated.

The overall structure of NmMafB2-CT2 has a kidney-like shape characterized by an RNase A fold and consists of two central β-sheets with three peripheral α-helices (Figs. 1B and C). The flat side of one β-sheet, which is composed of β1, β4 and β5, packs on the edge of the other central four stranded β-sheet, which consists of β2, β3, β6 and β7. The α2 and α3 helices flank the four-stranded β-sheet in opposite positions to each other and the α1 helix packs parallel with the four-stranded β-sheet. The structure of NmMafI2 consists of a four-stranded central β-sheet, one strand (β4′) of which is relatively short, and three helices. The α1′ and α2′ helices are arranged on the side of the central β-sheet and the α3′ helix is positioned on the other side of the central sheet. In the structure of the NmMafB2-CT2/NmMafI2 complex, the α2′ helix flanked by α1′ of NmMafI2 packs tightly against the shallow groove of NmMafB2-CT2 (Fig. 1B). The interaction between NmMafB2-CT2 and NmMafI2 is mediated by extensive hydrophobic and hydrogen bond interactions including eight salt bridges (Fig. 2A and Table 2). The residues in α1′ and α2′ helices and β3′ of NmMafI2 are mainly involved in interaction with NmMafB2-CT2, and the residues of NmMafB2-CT2 involved in the interaction with NmMafI2 are distributed broadly without involvement of the specific secondary structure of NmMafB2-CT2. Notably, the residues in α2′ of NmMafI2 contributes approximately 65% of the hydrogen bond and salt bridge interactions (Table 2). Upon complex formation, approximately 1110 Å^2^ of the binding interface becomes buried, which corresponds to approximately 17.0% and 17.5% of the surface areas of NmMafB2-CT2 and NmMafI2, respectively^25^.

**Table 2:** Interaction between NmMafB2-CT2 and NmMafI2.

NmMafI2		NmMafB2-CT2		Distance (Å)
Residue	Atom	Residue	Atom	
Hydrogen bonds				
Ile15′	O	Lys346	NZ	3.01
Ala16′	O	Lys346	NZ	3.10
Leu18′	O	Lys346	NZ	3.00
Thr62′	OG1	Asn377	ND2	2.84
Thr62′	OG1	His402	ND1	2.76
Ser66′	O	Asn414	N	2.78
Ser66′	O	Thr417	OG1	3.28
Ser66′	OG	Thr400	OG1	2.82
Met67′	O	Asn414	ND2	3.14
Glu74′	OE1	Arg375	N	3.01
Glu74′	OE2	Arg375	N	3.28
Ala75′	O	Arg375	NH1	3.01
Salt bridges				
Glu55′	OE2	Lys405	NZ	2.67
Glu58′	OE2	Arg375	NH2	2.76
Glu58′	OE1	Arg375	NE	2.96
Glu63′	OE1	Lys334	NZ	3.03
Glu63′	OE1	Lys346	NZ	3.05
Glu63′	OE2	Lys346	NZ	2.83
Glu70′	OE1	Lys420	NZ	3.37
Asp76′	OD1	Arg375	NH1	3.15

### Structural similarity between NmMafB2-CT2 and the RNase A fold

Because NmMafB2-CT2 does not show any sequence similarity to polypeptide with a known function, we performed structural homology searches with Dali server to obtain insights from structural similarity^26^. The search identified CdiA-CT^Ykris^ (PDB code: 5E3E) as the highest similar structure with a Z score of 4.4; this protein is the CDI toxin of *Yersinia kristensenii* and known as a novel member of the RNase A superfamily^20^. The structures of NmMafB2-CT2 and CdiA-CT^Ykris^ were overlapped with an rmsd of 2.3 Å over 66 Ca atoms (Fig. 1C). The next similar structure, with a Z score of 3.8, was MqsR from *E. coli* which belongs to RelE/YoeB ribonuclease family (PDB code: 3HI2)^27^; it was structurally similar with an rmsd of 2.6 Å over 63 Ca atoms. The amino acid sequences of the CdiA-CT^Ykris^ and MsqR have approximately 12% and 6% similarity, respectively, with NmMafB2-CT2. It has been shown that CdiA-CT^Ykris^ belongs to the RNase A superfamily with structural similarity to angiogenin and *Mus musculus* pancreatic RNase 1 and subsequent in vitro RNase assays^20^. The structures of NmMafB2-CT2 and RNase 1 also showed structural similarity, with a Z score of 3.0 Å and an rmsd of 3.9 Å over 76 Ca atoms (Fig. 1C) (PDB code: 3TSR)^28^. In addition, NmMafB2-CT2 displayed considerable similarity with colicin D (PDB code: 1V74)^29^, with a Z score of 3.5 and an rmsd of 2.9 Å over 70 Ca atoms, even though there was only approximately 6% sequence similarity. However, there are no β-sheet components in the structure of colicin D corresponding to β1, β4 and β5, which comprise central core β-sheets in the NmMafB2-CT2 structure (Supplementary Fig. 3).

### Structural diversity of immunity proteins binding to the RNase A fold

Structural homology searches with NmMafI2 using the Dali server identified the cytoplasmic domain of the zinc transporter CzrB (PDB code: 3BYP)^30^, which is thought to be Zn2 + /H + antiporter, as a best match with a Z score of 7.7 and an rmsd of 2.5 Å over 74 Ca atoms. The central β-sheet consisting of three relatively long strands (β1, β2 and β3) and two α-helices (α1 and α2) flanking on the one side of the central β-sheet overlapped quite well with each other, but there were no structural elements of the cytoplasmic domain of CzrB equivalent to the region (α3 and β4) of NmMafI2 (Supplementary Fig. 4). However, it seems that functional similarity based on a similar fold is limited because the cytoplasmic domain of CzrB, as part of the integrated transporter, is involved in Zn binding and transportation, whereas NmMafI2, as a soluble protein, functions as an inhibitor by specifically binding to its target, NmMafB2. For the inhibition of CdiA-CT^Ykris^, CdiI^Ykris^ uses a spherical structure consisting of eight α-helices to bind the shallow groove of CdiA-CT^Ykris^ that is used for RNA binding^20^. NmMafI2 uses a different overall architecture to fulfill a similar physical role by binding to the substrate binding site, thereby blocking substrate access.

### Catalytic sites

**Figure 3:**
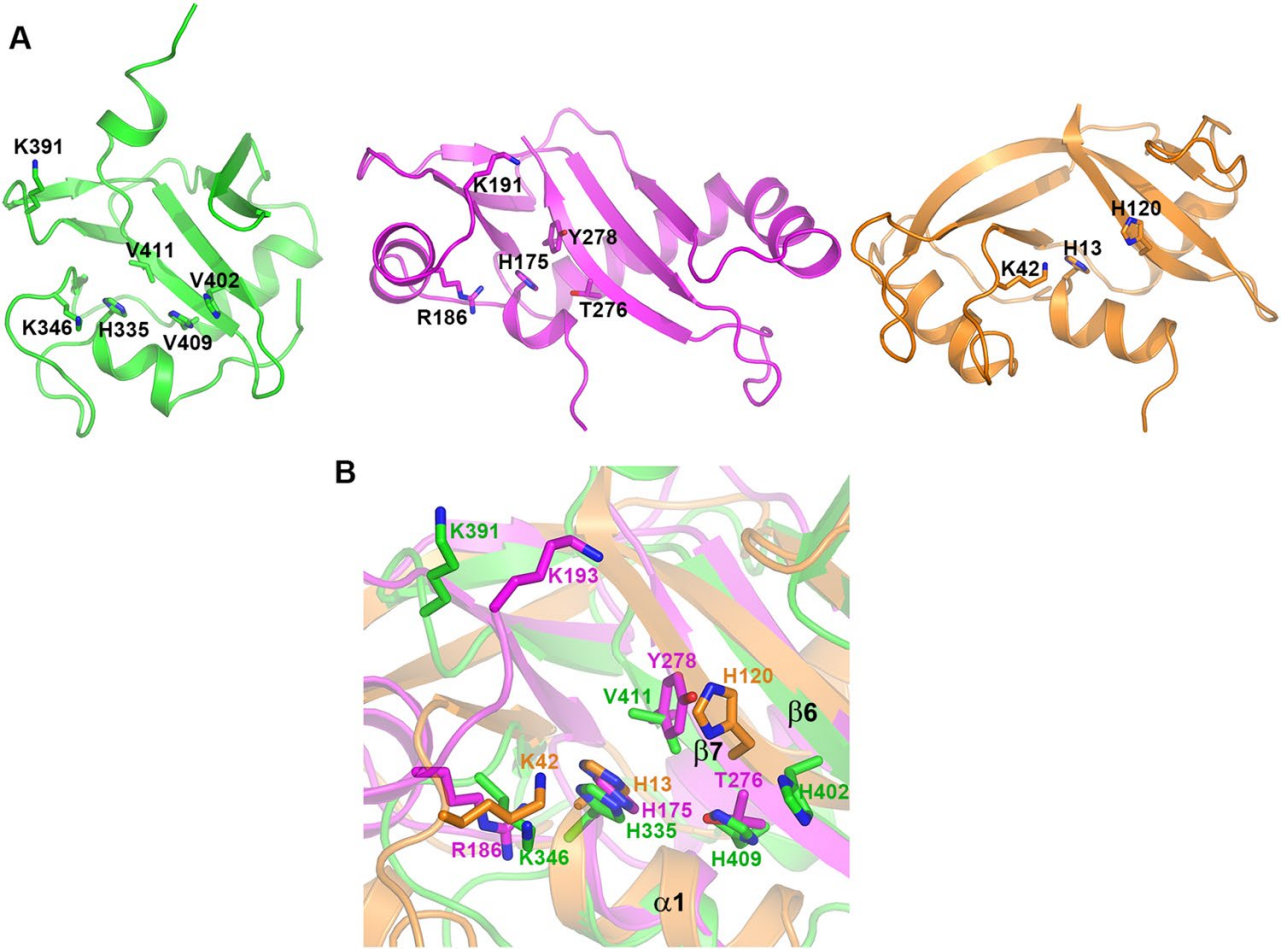
RNase fold of NmMafB2-CT2. (**A**) Ribbon diagram of NmMafB2-CT2 (left, green), CdiA-CT^Ykris^ (middle, magenta) and RNase 1 (right, orange). Catalytic residues are shown in a stick model. The view was rotated by ~ 90° vertically and clockwise from that of Fig. 1B. (**B**) Zoomed-in view of active sites. The stick model depicts residues of NmMafB2-CT2 superimposed on residues functioning as a base (H13 of RNase 1) or an acid (H120 of RNase 1 and T276 and Y278 of CdiA-CT^Ykris^), those providing stabilizing environment during catalysis.

Due to the similar fold of NmMafB2-CT2 and CdiA-CT^Ykris^, the latter of which was shown to be a member of the RNase A superfamily structurally and functionally, the structural superposition and structure-guided sequence alignment of NmMafB2-CT2 were performed with CdiA-CT^Ykris^ and mouse RNase 1. The nuclease sites of RNase A for RNA degradation consist of His12, Lys41 and His119^31,32^. His12 and His119 function as a base and acid for transphosphorylation reaction during RNA cleavage, respectively, and Lys41 is involved in stabilizing the intermediate of cyclic 2’-3’-cyclic nucleotides at the 5′ end^31,32^. His175 and Thr276/Tyr278 of CdiA-CT^Ykris^ correspond to His12 and His119 and Arg186/Ans191/Lys193 in the vicinity of Val192 correspond to Lys41 of RNase A (Fig. 3). When the structure of NmMafB2-CT2 was superimposed with those of CdiA-CT^Ykris^ and RNase 1, His335, thought to be a base, fits well with His13 of RNase 1 and His175 of CdiA-CT^Ykris^, suggesting that His335 of NmMafB2-CT2 could act as a base in the phosphotransferase reaction (Fig. 3). For the residue involved as an acid during the transphosphorylation reaction, there were no residues structurally matching His120 of RNase 1, but His402, His409 and Val411 of NmMafB2-CT2 were positioned near His120 of RNase 1 (Fig. 3B). Thr276 and Tyr278 of CdiA-CT^Ykris^ have been shown to be functionally equivalent to His120 of RNase 1 because these residues are found to be around His120 of RNase 1^20^. When comparing NmMafB2-CT2 and CdiA-CT^Ykris^, the His402, His409 and Val411 residues of NmMafB2-CT2 overlapped well with Thr276 and Tyr278 of CdiA-CT^Ykris^ (Fig. 3B)*.* Therefore, His402, His409 and Val411 of NmMafB2-CT2 are likely act as acids for transphosphorylation reaction during RNA cleavage. Lys42 of RNase 1 is involved in stabilizing the intermediate during the transphosphorylation reaction^31^. Lys346 and Lys391 of NmMafB2-CT2 likely functionally correspond to this residue because they are positioned near Lys42 of RNase 1 and Arg186 and Lys193 of CdiA-CT^Ykris^, which have shown to be important for RNA cleavage^20,32^.

### Interaction interface between NmMafB2-CT2 and NmMafI2

The similar fold between NmMafB2-CT2 and RNase A superfamily members, such as RNase 1 and CdiA-CT^Ykris^, suggests that NmMafB2-CT2 has a positively charged surface for recognizing negatively the charged phosphate backbone of RNA. The surface charge distribution of NmMafB2-CT2 shows three positively charged regions that surround a central shallow groove with a catalytic base residue, H335, at the bottom. The first region is formed by Lys391 and Lys420, the second region by Arg340, Lys334, and Lys346, and the third region by Lys372, Arg375 and Lys405 (Fig. 2B). The central groove of NmMafB2-CT2 fits one side of NmMafI2, which is characterized by a negatively charged surface consisting of residues from α2′ (Glu55′, Glu58′ and Glu63′), the loop connecting α2′ and β3′ (Glu70′) and β3′ (Glu74′ and Asp76′) (Fig. 2B). All positively charged residues of NmMafB2-CT2 in the complex interface are involved in salt-bridge interactions with NmMafI2, with the exception of Arg340, Lys372 and Lys391 (Fig. 2A and Table 2). Lys391 forms a hydrophobic interaction with Val11 of NmMafI2, but K372 and K340 are not involved in any contact (Fig. 2A). All negatively charged residues of NmMafI2 in the complex interface contribute to salt-bridge interactions with NmMafB2-CT2, except that Glu74 of NmMafI2 forms hydrogen bonds with NmMafB2-CT2 (Fig. 2A and Table 2). These results indicate that complementary charge interactions are the main driving force for the formation of the complex between NmMafB2-CT2 and NmMafI2, and that NmMafI2 functions as an inhibitory regulator protein by blocking RNA from accessing the enzymatic cleavage site because it binds the substrate binding site of NmMafB2-CT2.

### Binding affinity between NmMafB2-CT2 and NmMafI2

**Figure 4:**
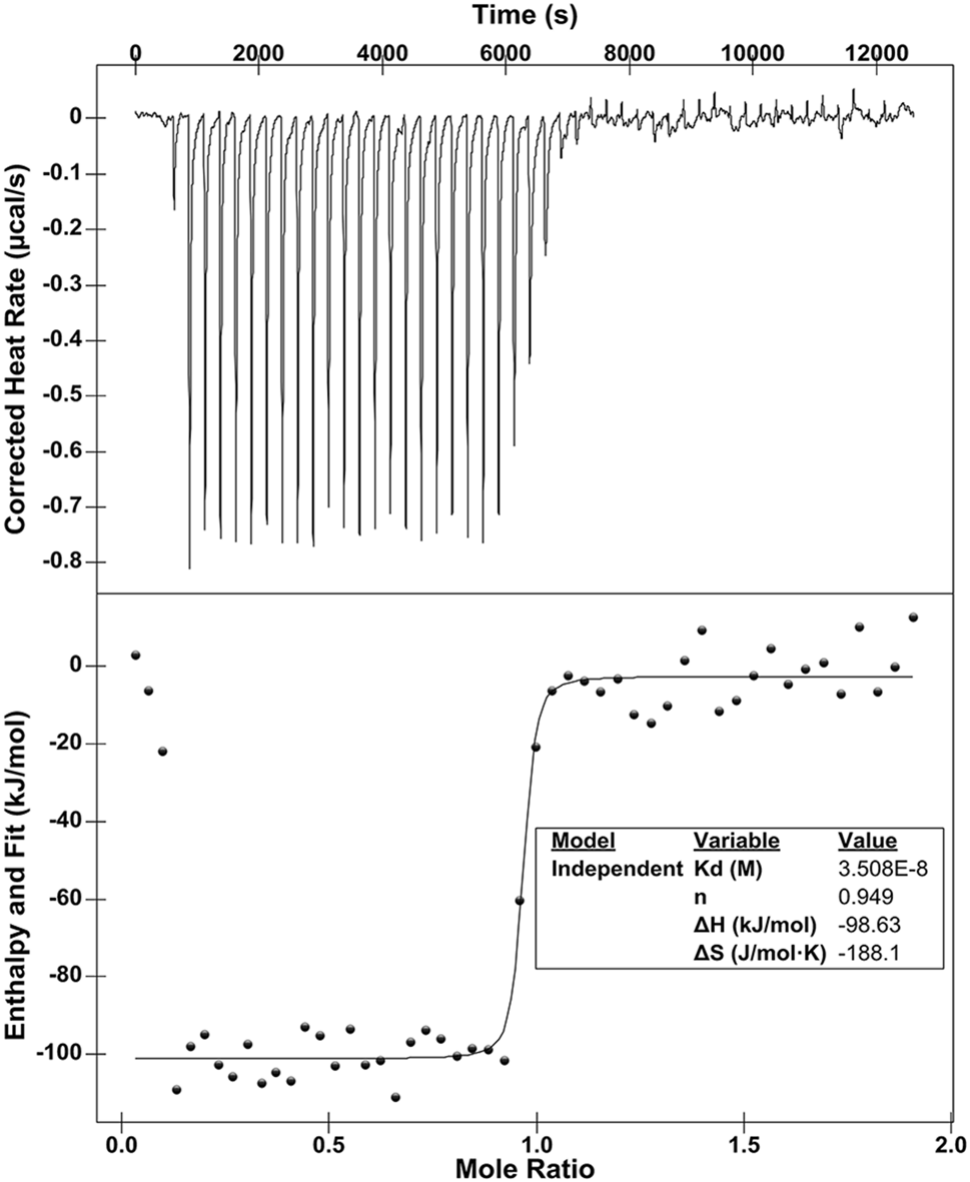
Interaction between NmMafB2-CT2 and NmMafI2. Isothermal titration calorimetry (ITC) for measuring the binding affinity of NmMafI2 to NmMafB2-CT2. The titration of NmMafB2-CT2 (0.2 mM) was performed by 50 injections of 1.3 mM NmMafI2.

To determine the binding affinity between NmMafB2-CT2 and NmMafI2, the toxin domain was titrated with immunity protein and the released heat was measured by nano-isothermal calorimetry. NmMafB2-CT2 was purified by refolding after isolation from the NmMafB2-CT2/NmMafI2 complex and used for isothermal calorimetry. The dissociation constant of the NmMafB2-CT2/NmMafI2 complex was measured to be ~ 40 nM (Fig. 4). The binding constants of NmMafB2-CT2 was comparable with that of CdiA-CT^Ykris^, which has an approximately 40 nM dissociation constant for CdiI^Ykris^^20^. The stoichiometry was calculated to be with approximately 1, which is consistent with the 1:1 interaction in the crystal structure (Figs. 1 and 4).

### In vitro enzymatic assay

**Figure 5:**
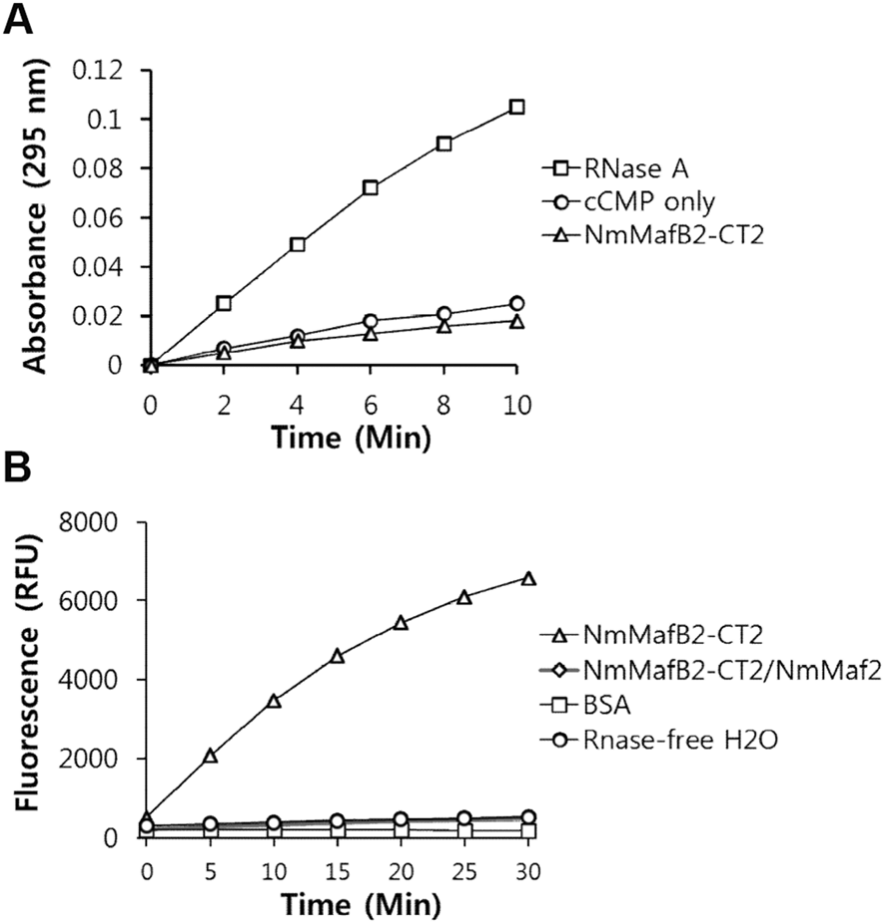
Enzymatic assay of NmMafB2-CT2 in vitro. (**A**) Phosphodiester hydrolysis activity assay. The hydrolysis of cCMP was analyzed by measuring the increase in absorbance at 296 nm for 10 min. RNase A and cCMP were also included as the positive and negative controls, respectively. (**B**) RNase assay. The RNA cleavage activities were measured with a fluorophotometer using RNaseAlert fluorescent substrate at Ex/Em = 485/530 nm at 37 °C for 30 min. NmMafB2-CT2/NmMafI2 was the purified protein used to obtain NmMafB2-CT2. BSA: bovine serum albumin; RFU: relative fluorescence unit.

The conserved structural fold of NmMafB2-CT2, CdiA-CT^Ykris^ and RNase 1 suggests that NmMafB2-CT may also have ribonuclease activity. Ribonuclease assays using the RNaseAlert Lab Test Kit (Invitrogen), which emits a fluorescence when RNA oligonucleotides tethering a fluorophore and a quencher is cleaved, showed that NmMafB2-CT2, which was refolded after isolation from the NmMafB2-CT2/NmMafI2 complex, degraded the RNA oligonucleotide (Fig. 5B). However, the NmMafB2-CT2/NmMafI2 complex, which was used for purifying NmMafB2-CT2, did not degraded the RNA substrate. As negative controls, RNase-free water and BSA did not degrade the RNA substrate (Fig. 5B). RNase A has transphosphorylation activity as well as the hydrolysis activity of the nucleoside 2',-3'-cyclic phosphodiester bond, which is the reverse of the transphosphorylation activity that occurs during RNA degradation^32^. Therefore, the hydrolysis assay of the phosphodiester bond can be used to determine whether a polypeptide has ribonuclease activity. The hydrolysis activity of NmMafB2-CT2 was investigated spectroscopically using cytidine 2',3' cyclic monophosphate (cCMP). RNase A as a positive control was also included for the hydrolysis assay. RNase A hydrolyzed cCMP, but NmMafB2-CT2 did not have hydrolysis activity (Fig. 5A). The inclusion of divalent ions such as Mg^2+^, Co^2+^, Ni^2+^ and Zn^2+^ or using more NmMafB2-CT2 had no effect on the hydrolysis activity (data not shown). These results suggest that in contrast to the known RNase A family enzymes and CdiA-CT^Ykris^, NmMafB2-CT2 does not hydrolyze the the nucleoside 2',-3'-cyclic phosphodiester bond, rather NmMafB2-CT2 has only transphosphorylation activity for degrading RNA. It is unlikely that the loss of hydrolysis activity was due to incorrect folding during the refolding NmMafB2-CT2 isolated from the denatured solution because the binding affinity of the refolded NmMafB2-CT2 to NmMafI2 was comparable with that of CdiA-CT^Ykris^ to CdiI^Ykris^ and the refolded NmMafB2-CT2 had ribonuclease activity (Figs. 4 and 5A).

### Potential functional sites

**Figure 6:**
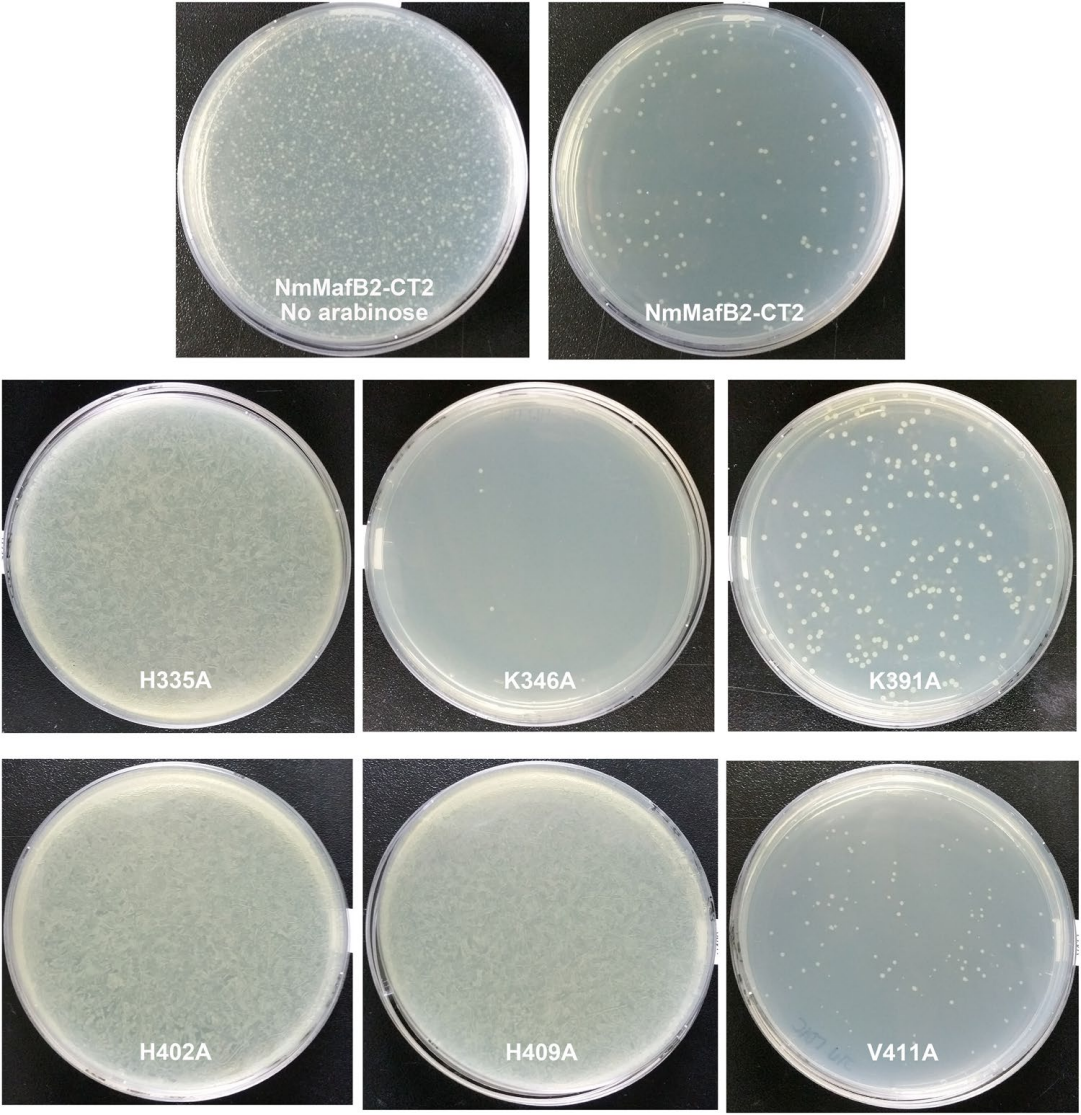
Toxicity of NmMafB2-CT2 in *E. coli*. The toxic activity of NmMafB2-CT2 and its mutants was assessed by counting the number of colonies. Overnight cultures of BL21 Star (DE3) transformed with the NmMafB2-CT2 or variants were diluted to an OD600 of 0.1 in fresh LB medium, and 0.2% L-arabinose was added to initiate toxin expression. After a further 6 h of incubation at 37 °C, the cultures were appropriately diluted and spread on agar plate and the plates were incubated at 37 °C overnight.

Because the toxin domains are toxic when expressed in heterologous expression systems such as *E. coli* BL21 (DE3), the toxin domain was obtained by refolding after being purified in complex with its cognate immunity protein^20,21,33^. These results suggest that it is possible to determine the potential functionally functional important residues (for example, the catalytic or substrate recognizing residues in the case of RNase A) by measuring the growth curve after initiating expression of a polypeptide with toxin activity. Accordingly, to determine the functional important residues of NmMafB2-CT2 that were identified by the fold similarity analysis, alanine mutants were generated and expressed under the control of L-arabinose. And then the toxic effect of each mutant on cell viability was determined by counting the number of clones that grew from the cell cultures on LB plates. The cell viability of cells expressing wild-type NmMafB2-CT2 was markedly reduced compared to that of the same culture in which no L-arabinose was added for expression, suggesting that NmMafB2-CT2 exhibits toxic activity, probably owing to RNA-degrading enzymatic activity (Fig. 6). In the case of the H335A mutant, which was thought to have a defect in the residue functioning as a general base, a reduction in cell viability was not observed, suggesting that H335 plays a catalytically important role, probably as a general base during the transphosphorylation reaction. This was consistent with the fact that the H335A mutant was readily expressed and purified without the inclusion of an immunity protein to neutralize of toxic activity during expression and purification (data not shown). In the case of the H402A and H409A mutants, which were thought to have defects in functions similar to those of H120 of mouse RNase 1 and T276 and Y278 of CdiA-CT^Ykris^, the cell viability was similar to cells expressing the H335A mutant, suggesting that H409 and H402 are catalytically important residues, probably by acting as acids during the transphosphorylation reaction. In contrast, the V411A mutant showed similar viability to that of cells expressing wild-type NmMafB2-CT2, indicating that V411 is dispensable, although V411 is structurally overlaid with Y278 of CdiA-CT^Ykris^ and H120 of mouse RNase 1. In the case of structural overlap with CdiA-CT^Ykris^, Y278 is structurally equivalent to V411 of NmMafB2-CT2. However, it seems that the absence of atoms such as oxygen in the valine residue restricts the role of V411 as an acid during the reaction. For the K346A and K391A mutants with defects that are probably in a similar role to that of K42 of mouse RNase 1, the cell viability was similar to that of cells expressing wild-type NmMafB2-CT2, indicating that K346 and K391 are not catalytically important residues. Collectively, the results indicate that H335 functions as a general base, H402 and H409 function as acids, but K346 and K391 are not catalytically important, during the RNA transphosphorylation reaction.

## Conclusion

In this study, we determined the X-ray crystal structure of the complex formed by the toxin domain of MafB2_MGI-2B16B6_ and the corresponding immunity protein, MafI2_MGI-2B16B6_, from *N. meningitidis* B16B6. The toxin domain of MafB2_MGI-2B16B6_, adopts the RNase A fold and the subsequent biochemical analysis showed that the toxin domain of MafB2_MGI-2B16B6_, has ribonuclease activity. These data provide structural and biochemical evidence that the toxic activity of MafB2_MGI-2B16B6_ from *N. meningitidis* is based on non-specific RNA degrading activity.

## Materials and methods

### Gene cloning

The codon optimized genes encoding the toxin domain (Ala270–the C-terminus) of MafB2_MGI-2B16B6_, hereafter referred as NmMafB2-CT, and the corresponding immunity protein, MafI2_MGI-2B16B6_, hereafter referred to as NmMafI2 from *N. meningitidis* B16B6 were synthesized with sequences of restriction enzyme sites for cloning (IDT). During the gene synthesis of NmMafB2-CT, a single point mutation (Gly to Arg) at residue 376 was introduced inadvertently. The G376R mutant was used for the structural determination. The G376R mutant gene of NmMafB2-CT was subcloned into a modified pET28a (pSKB3) vector with a His_6_-tag on the N-terminus and a TEV cleavage site using *NdeI* and *EcoRI* restriction enzyme sites. The gene of NmMafB2-CT with the original amino acid sequences was then obtained with site directed mutagenesis using two primers (Table S1). The NmMafI2_MGI-2B16B6_ gene was subcloned into the pET21d vector using *NcoI* and *EcoRI* or *XhoI* restriction enzymes in such a way that NmMafI2 had no tag or C-terminal His6-tag, respectively, when expressed. A short version of the toxin domain (Ile323–the C-terminus) of MafB2_MGI-2B16B6_, hereafter referred to as NmMafB2-CT2, was designed because the SDS-PAGE analysis of crystal samples revealed that NmMafB2-CT was degraded during crystallization (Supplementary Fig. 1). The N-terminal end of NmMafB2-CT2 was chosen based on the end of the electron density map of the structure of NmMafB2-CT2/NmMafI2 complex. NmMafB2-CT2 was cloned into pET28a and pET21d vectors using *NcoI* and *EcoRI* restriction enzymes for no His6-tag expression or *NcoI* and *XhoI* for His6-tag expression with PCR product amplified using pSKB3-NmMafB2-CT as a template and two primers (Table S1).

### Mutagenesis

Site-directed mutagenesis was performed with *Pfu* DNA polymerase using a protocol based on the QuickChange II site-directed mutagenesis kit (Agilent Technologies). The forward and reverse primers for each mutant are listed in Table S1.

### Purification of the NmMafB2-CT/NmMafI2 complex

The two plasmids, the G376R mutant of NmMafB2-CT with a TEV-cleavable N-terminal 6xHis-tag and NmMafI2 with no affinity tag, were coexpressed in *E. coli* BL21 Star (DE3) cells (Invitrogen). The NmMafB2-CT/NmMafI2 complex was expressed by adding isopropyl β-D-1-thiogalactopyranoside (IPTG) at a final concentration of 0.5 mM once the OD_600nm_ of cells grown at 37 °C in LB medium was about ~ 1.0 and incubating the cultures for 15 h at 20 °C. The cell cultures were harvested by centrifugation at 5000 g at 4 °C. The cell pellet was resuspended in buffer A (20 mM Tris–HCl pH 8.0, 0.2 M NaCl, 0.1 mM EDTA, 2 mM β-mercaptoethanol (β-ME) and 30 mM imidazole) and lysed by sonication. The lysate was centrifuged at 15,000 g for 30 min and the supernatant was loaded onto a 5 ml HisTrap crude column (Cytiva) equilibrated with buffer B (20 mM Tris pH 8.0, 0.2 M NaCl, 2 mM β-ME, 30 mM imidazole). The complex was eluted using a linear gradient of 30 to 500 mM imidazole. TEV protease at a target protein to protease ratio of 50:1 (w/w) was added to remove the N-terminal 6xHis-tag during dialysis at 4 °C overnight with buffer C (10 mM Tris–HCl pH 8.0, 50 mM NaCl and 2 mM β-ME). The NmMafB2-CT/NmMafI2 complex without the His-tag was then purified by collecting the flow through fractions on a 5 ml HisTrap crude column (Cytiva) equilibrated with buffer B. Finally, the complex was purified with a Superdex 200 (Cytiva) column equilibrated in 10 mM Tris–HCl pH 8.0, 100 mM NaCl and 2 mM β-ME. The absorbance at 280 nm was measured, and the protein concentration was calculated using the extinction coefficient of 24,410 M^-1^ cm^-1^ obtained from ProtParm available at the ExPASy web site (http://web.expasy.org/protparam). The selenomethionine (Se-Met) labeled NmMafB2-CT/NmMafI2 complex was expressed using the PASM autoinduction medium^34^ and purified with the same protocol as the native protein.

### Crystallization and data collection

Initial crystallization conditions were screened in 96-well MRC Crystallization Plates (Molecular Dimension) using commercial crystallization kits such as Crystal Screen 1 and 2, Index and Salt from Hampton Research (California, USA) and Wizard Classic 1 and 2 from Regaku Reagents (Washington, USA). The G376R mutant of the NmMafB2-CT/NmMafI2 complex (0.8 µl) at a concentration of 10 mg/ml was mixed with anequal volume of reagent with a Mosquito robot (SPT Labtech), and then the drops were equilibrated against 70 µl of reservoir solution at 22 °C. The initial condition was optimized using the hanging-drop vapor-diffusion method with VDX plates (Hampton Research). The final plate-shaped crystals for X-ray diffraction were obtained in the wells containing 0.25—0.3 M magnesium formate and 25–30% polyethylene glycol (PEG) 3350 (Supplementary Fig. 1). For X-ray diffraction, a single crystal obtained by breaking a relatively large crystal with a micro needle was soaked briefly in cryosolution (0.3 M magnesium formate, 30% PEG 3350 and 25% glycerol) and then flash-frozen directly in a liquid nitrogen stream at 100 K using a sample loop. Crystals of Sel-Met labeled complex were produced under similar conditions. A native dataset consisting of 360 images were collected on an ADSC Q270 CCD detector at beamline 7A of the Pohang Light Source II (PLS II) at Pohang Accelerator Laboratory (PAL, Pohang, Korea) with 1° oscillations at a wavelength of 0.9794 Å. An SAD dataset wase collected with the same scheme as the native data at the selenium edge energy (0.97917 Å). The data were indexed and scaled with the HKL-2000 software package^35^. The crystallographic data statistics are summarized in Table 1.

### Structure determination

The Autosol wizard in the *PHENIX* package was used to determine the selenium sites and electron density modification of the initial map with the SAD dataset^36^*.* Automatic model building was then carried out with the AutoBuild wizard in the *PHENIX* package^37^ using the density modified map. The missing parts of the model were traced manually using *Coot*^38^. The model was then modified by iterative manual minor adjustment and refinement using *Coot* and *PHENIX*, respectively. The solvent model was built automatically during *PHENIX* refinement, which was further checked with manual inspection using *Coot* with the aid of a sigma-A weighted Fo-Fc map. The refinement statistics are summarized in Table 1. All figures of the molecular model were produced with *PyMol* (www.pymol.org).

### Expression and purification for biochemical and biophysical studies

The two plasmids with different antibiotic resistances, one encoding NmMafB2-CT2 with no tag and the other encoding NmMafI2 with a C-terminal 6xHis-tag, were coexpressed. The complex was purified using a His-tag column with the same protocol as that for the NmMafB2-CT/NmMafI2 complex. NmMafB2-CT2 was isolated by collecting the flow through fractions using a 5 ml HisTrap crude column (Cytiva) in denaturing buffer (20 mM Tris pH 8.0, 200 mM NaCl, 6 M guanidine-HCl and 30 mM imidazole) and then refolded by dialysis against 20 mM Tris pH 8.0, 100 mM NaCl and 2 mM β-ME overnight. After further dialysis aginst with 20 mM HEPES pH 7.5, 50 mM NaCl, 2 mM β-ME, NmMafB2-CT2 was purified further by linear NaCl gradient elution from a 5 ml Hitrap-SP column (Cytiva), that was equilibrated with 20 mM HEPES pH 7.5, 50 mM NaCl, 0.5 mM EDTA, and 2 mM β-ME. Finally, NmMafB2-CT2 was dialyzed against 10 mM Tris–HCl pH 8.0, 100 mM NaCl and 2 mM β-ME. NmMafI2 with a C-terminal 6xHis-tag was expressed and purified with the same protocol as that for the NmMafB2-CT/NmMafI2 complex omitting the steps of removing the N-terminal 6xHis-tag. The protein concentration was determined using the following extinction coefficient at 280 nm calculated by ProtParam program: NmMafB2-CT2, 4470 M^-1^ cm^-1^; NmMafI2, 18,450 M^-1^ cm^-1^.

### Isothermal titration calorimetry

The affinity between NmMafB2-CT2 and NmMafI2 was measured at 20 °C using a Nano ITC calorimeter (low volume cell 190 μl; TA instruments). All samples were prepared in the same buffer (10 mM Tris–HCl pH 8.0, 100 mM NaCl and 2 mM β-ME). Titration was performed by injecting 1 μl of NmMafI2 (~ 1.3 mM) into the cell containing NmMafB2-CT2 (~ 0.2 mM) 50 times at 250 s intervals. The NanoAnalyze program (TA Instruments) was used to calculate the interaction parameters after fitting the data with the independent binding sites model. The titration curve used for the program analysis was baseline-corrected with the buffer used in the titration.

### Enzyme assay

In vitro hydrolysis activity was assessed spectroscopically in 20 mM Tris pH 7.5 and 150 mM NaCl buffer using 3 mM cCMP. The cCMP solution was incubated with 20 µM NmMafB2-CT2 in a final volume of 80 µl at room temperature. And then the absorbance at 296 nm was measured for 10 min. As a positive control, bovine pancreatic RNase A at a concentration of 20 µM was included in the hydrolysis activity assay. To examine the dependency of metal atoms, metal atoms at a concentration of 0.2 mM was included in the reaction buffer. RNase activity was measured by a fluorophotometer using an RNaseAlert Lab Test Kit (Invitrogen). The reaction mixtures in the final 50 µl volume containing 10 µg of protein were prepared in black 96-flat well plates. The fluorescence intensity was measured at 37 °C for 30 min using a Tecan Spark microplate reader (Tecan) with emission (Em) at 530 nm and excitation (Ex) at 485 nm with a 20 nm bandwidth for both wavelengths. As negative controls, BSA and RNase-free H_2_O were also included. All measurement were repeated three times.

### Toxicity assay in *E. coli*

All mutants were generated with the protocol described above using pET28a-MafB2-CT2 as a template and each forward and reverse primers (Table S1). Each mutant was amplified by PCR using the forward and reverse primers (Table S1), and subcloned into pBAD33.1^39^ using *NdeI* and *HindIII* restriction enzyme sites in such a way that no His-tag was expressed. The mutants were transformed into BL21 Star (DE3) cells, and each clone was cultured in 3 ml of LB medium containing 50 µg/ml chloramphenicol and 0.2% L-glucose at 37 °C overnight. The cultures were then diluted into 3 ml of fresh LB medium containing 50 µg/ml chloramphenicol and 0.2% L-arabinose at an OD600 of 0.1 and cultured further for 6 h at 37 °C. Finally, the cultures were diluted and then plated on LB plates containing 50 µg/ml chloramphenicol to check cell viability.

## Supplementary Information

Supplementary Information.

## Data Availability

The atomic coordinates and structure factors were deposited in the Protein Data Bank using accession number 8HHJ.
