# Supplementary material for: Structural basis for the toxic activity of MafB2 from maf genomic island 2 (MGI-2) in N. meningitidis B16B6
Source: Sci Rep. 2023 Feb 27;13:3365. doi: 10.1038/s41598-023-30528-9 (PMC9970974; doi:10.1038/s41598-023-30528-9)
Supplement: Supplementary file 1 — Supplementary Information. [file 41598_2023_30528_MOESM1_ESM.doc]

**Supplementary information**

**Structural basis for the toxic activity of MafB2 from maf genomic island 2 (MGI-2) in *N. meningitidis* B16B6**

So Hyeon Park, Sun Ju Jeong and Sung Chul Ha*

Beamline Department, Pohang Accelerator Laboratory, Pohang University of Science and Technology, Pohang, Gyeongbuk 37673, Republic of Korea

*Correspondence: scha2@postech.ac.kr


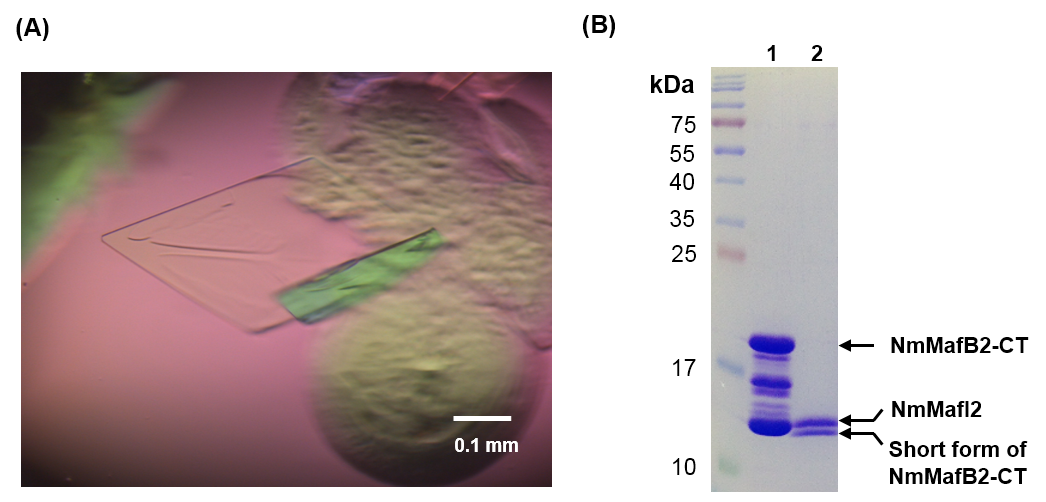


**
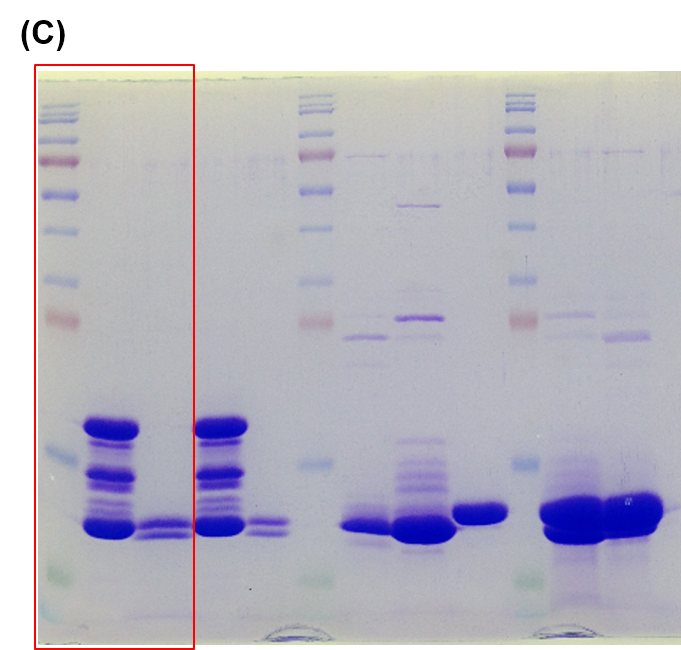
**

**Supplementary Figure 1. Complex between NmMaf2-CT and NmMafI2.** (A) Optimized crystals of NmMafB2-CT in complex with NmMafI2. Thin plate crystals formed under crystallization conditions of 0.25–0.3 M magnesium formate and 25–30% PEG 3350. (B) SDS-PAGE analysis of crystals of NmMafB2-CT in complex with NmMafI2 (lane 1, sample used for crystallization; 1ane 2, sample obtained by dissolving several optimized crystals). The standard molecular weights and the positions of NmMafB2-CT and NmMafI2 are labeled. During crystallization, NmMafB2-CT appeared to be degraded to the short form of NmMafB2-CT, with a molecular weight slightly lower than that of NmMafI2. (C) Uncropped SDS-PAGE gel. The lanes in the red box were used in Supplementary Fig. 1B.

**Supplementary Figure 2. Alignments of MafB2 and MafI2 from two different strains of *N. meningitidis*.** (A) Alignment of MafB2_MGI-2B16B6_ from *N. meningitidis* B16B6 with MafB2_MGI-2NEM8013_ from from *N. mningitidis* NEM8013 using ClustalW. The non-identical and insert residues are in red. The sequence encompassing NmMafB2-CT2 is boxed with green. (B) Alignment of MafI2_MGI-2B16B6_ from *N. meningitidis* B16B6 with MafI2_MGI-2NEM8013_ from *N. mningitidis* NEM8013 using ClustalW. The non-identical residues are in red.


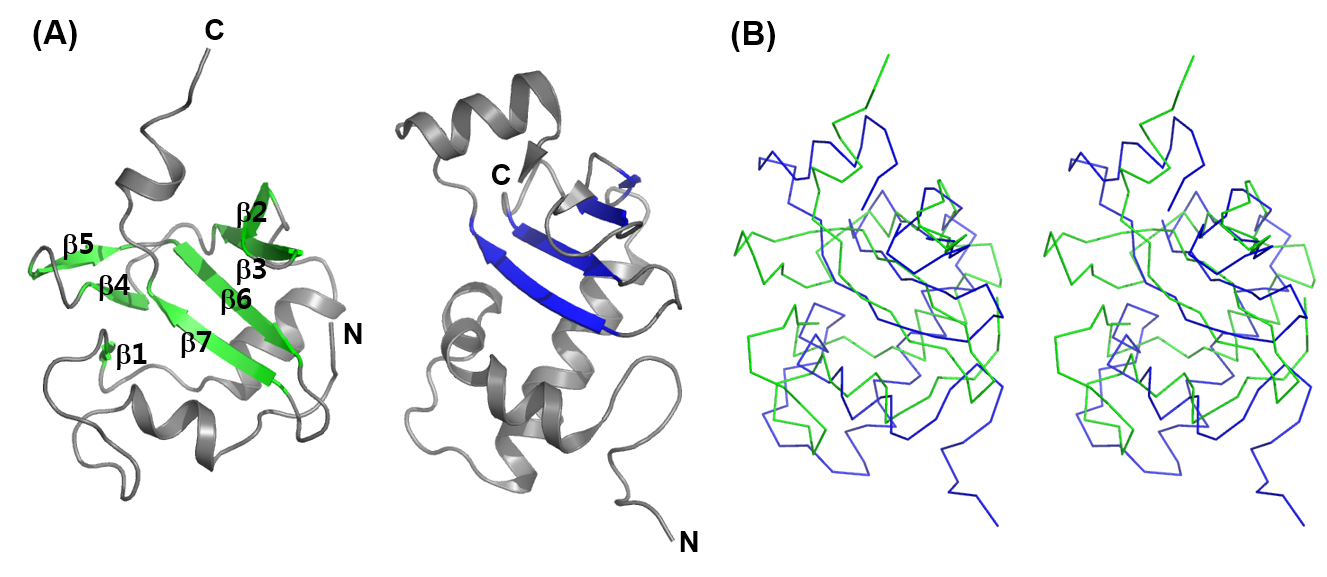


**Supplementary Figure 3. Structural comparison between NmMafB2-CT2 and colicin D** (A) Ribbon diagram of NmMafB2-CT2 (left) and colicin D (right, PDB code: 1V74) with only central β-strands colored green and blue, respectively. N- and C-termini and β-strands of NmMafB2-CT2 are labeled. In the structure of colicin D, no β-sheet was formed that was equivalent to β1, β4 and β5 in the structure of NmMafB2-CT2. (B) Stereoview of theoverlap between NmMafB2-CT2 (green) and colicin D (blue).


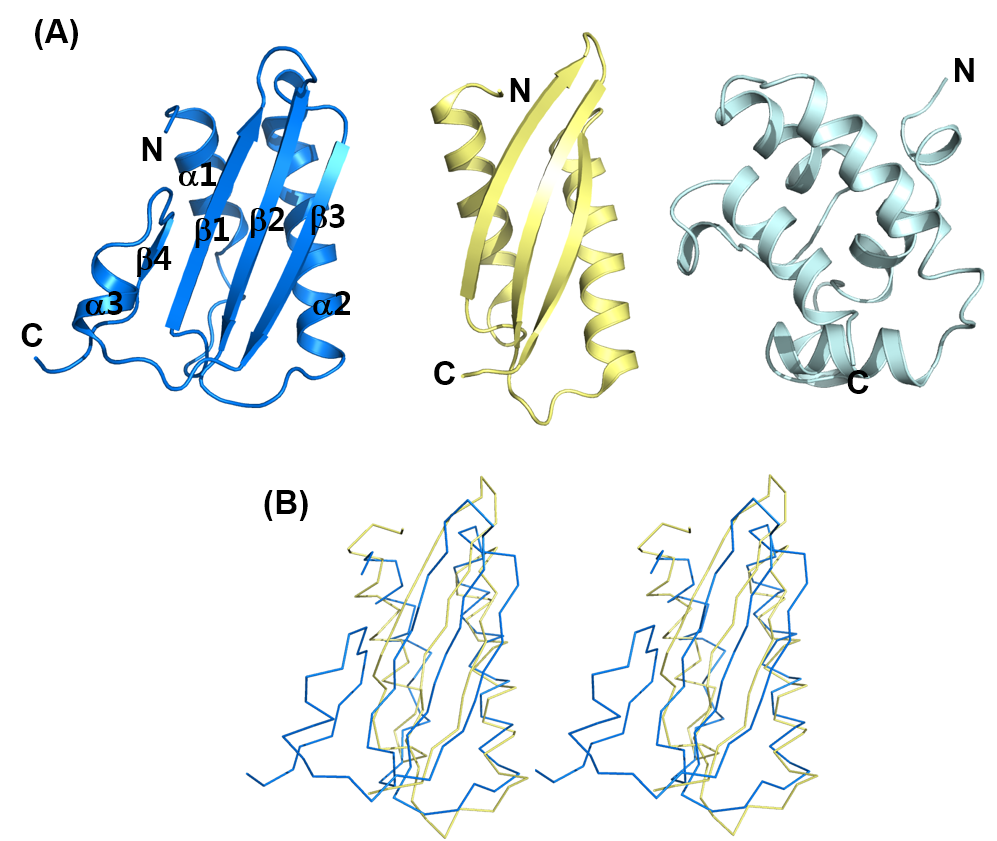


**Supplementary Figure 4. Structural comparison of NmMafI2 with the cytoplasmic domain of CzrB and CdiI^Ykris^.** (A) Ribbon diagram of NmMafI2, the cytoplasmic domain of CzrB (middle, PDB code: 3BYP) and CdiI^Ykris^ (right, PDB code: 5E3E). N- and C-termini and the secondary structures of NmMafI2 are labeled. The orientation of CdiI^Ykris^ was based on the position of CdiA-CT^Ykris^ (not shown here) that was positioned similarly to NmMafB2-CT2. (B) Stereoview of overlap between NmMafI2 (blue) and the cytoplasmic domain of CzrB (yellow). In the cytoplasmic domain of CzrB, there is no part equivalent to that comprising α3 and β5 of in the structure of NmMafI2

**Table S1.** **Primers used in this study.**

| **Name** | **Sequences (5' to 3')** |
| --- | --- |
| MafB2CT-R376G-foward | GTGATTTCAAAAGGCGCCCGTGGAAACCCCGTGGTAGACTTCGGA |
| MafB2CT-R376G reverse | TCCGAAGTCTACCACGGGGTTTCCACGGGCGCCTTTTGAAATCAC |
| MafB2CT2-foward | CG CCATGG GC ATCGGCACCAAAATCCACGATGGTGCGC |
| MafB2CT2-reverse | CGGAATTCACTCGAGTTGCACTTTTTTTATGGTTTTTGGATTGGC |
| H335A-foward | GATGGTGCGCAGGGCAAAGCCATCAGCGGACACAGAAACTAC |
| H335A-reverse | GTAGTTTCTGTGTCCGCTGATGGCTTTGCCCTGCGCACCATC |
| K346A-foward | CAGAAACTACATCGAGGGGGCATCTACGCTGAACCAGAAC |
| K346A-reverse | GTTCTGGTTCAGCGTAGATGCCCCCTCGATGTAGTTTCTG |
| K391A-foward | CCCATAGGGTCTGATGGCGCATCTGGCTTATCGACGAA |
| K391A-reverse | TTCGTCGATAAGCCAGATGCGCCATCAGACCCTATGGG |
| H402A-foward | GAACTTTGGAACGATTGCCTCTGGTAAAAACGGAG |
| H402A-reverse | CTCCGTTTTTACCAGAGGCAATCGTTCCAAAGTTC |
| H409A-foward | CACTCTGGTAAAAACGGAGTTGCCATAGTACCGGCCAATCC |
| H409A-reverse | GGATTGGCCGGTACTATGGCAACTCCGTTTTTACCAGAGTG |
| V411A-foward | GGTAAAAACGGAGTTCACATAGCCCCGGCCAATCCAAAAACC |
| V411A-reverse | GGTTTTTGGATTGGCCGGGGCTATGTGAACTCCGTTTTTACC |
| pBAD33.1-foward | CGATACCATATGATCGGCACCAAAATCCACGATGGTGCGC |
| pBAD33.1-reverse | GCGAAGCTTTTATTGCACTTTTTTTATGGTTTTTGGATTGGC |
|  |  |

The restriction and mutated sites are underlined.
